# Genotoxicity, Bioactivity and Clinical Properties of Calcium Silicate Based Sealers: A Literature Review

**DOI:** 10.22037/iej.v12i4.17623

**Published:** 2017

**Authors:** Farnaz Jafari, Sanaz Jafari, Paria Etesamnia

**Affiliations:** a *Department of Endodontics, Dental School, Tabriz Branch, Islamic Azad University, Tabriz, Iran; *; b * Department of Orthodontics, Dental School, Ilam University of Medical Sciences;*; c * Department of Operative Dentistry, Dental School, Tabriz Branch, Islamic Azad University, Tabriz, Iran*

**Keywords:** Bioactive Materials, Genotoxicity, Mineral Trioxide Aggregate, Root Canal Sealers, Tricalcium Silicate

## Abstract

An ideal endodontic sealer should have many properties such as excellent seal after setting, dimensional stability, slow setting time to ensure sufficient working time, insolubility to tissue fluids, adequate adhesion with canal walls and biocompatibility. Genotoxicity is one of the important factors that influence biocompatibility of an endodontic sealer. This literature review was conducted to survey the genotoxicity, bioactivity and clinical perspectives of calcium silicate based sealers. We searched PubMed using appropriate MeSH keywords. Also a hand search was conducted in the related journals. Sixty eight articles were assessed finally. Genotoxicity and acute inflammation were high in calcium silicate based sealers. Both resin-based and calcium silicate based sealers caused perceptible tooth discoloration. There were controversies regarding the fracture resistance, apical patency and retreatability of different sealers. Clinical properties of calcium silicate-based sealers are also outlined.

## Introduction

Root canal sealers play an important role in the success of root canal treatment by decreasing the amount of sealer used and having a good adaptation of sealer to root dentin [1]. Sealers have different categories based on their fundamental chemical compounds including zinc oxide eugenol, calcium hydroxide (CH), glass ionomer, silicone, resin and bioceramic based sealers [2-6]. Advancements in bioceramic technology have revolutionized endodontic material science by enhancing the treatment outcome for patients. This class of dental materials claims excellent biocompatibility with high osteo-conductivity that makes them ideal for endodontic application. Due to the relative biological and technical importance of sealers, their genotoxicity, bioactivity and discoloration potential have been the subject of considerable attention. Bioceramic-based sealers are categorized into two groups of calcium silicate based sealers (Mineral trioxide aggregate (MTA)-based and non-MTA-based sealers) and calcium phosphate-based sealers [7]. This article focuses on MTA-based sealers. Bioactive materials, such as glass and calcium phosphate, interact with the surrounding tissue to encourage the growth of more durable tissues [8]. 

Calcium silicate based sealers were introduced with the aim of combining the physicochemical properties of a root canal sealer [9] with the benefit of MTA’s biocompatibility and bioactivity. This review was conducted to assess and summarize biologic and and clinical properties of calcium silicate based sealers based in the available literature.

## Materials and Methods

A review of the literature from peer-reviewed journals published in English was performed by using electronic and hand-searching methods. Appropriate MeSH terms and keywords including “Tricalcium silicate”, “Root canal sealers”, “Material characterization”, “Hydration”, “Mineral trioxide aggregate (MTA)”, “Bioactive materials”, “calcium silicate based sealers”, “MTA Fillapex”, “genotoxicity”, “bioactivity”, “fracture resistance”, “retreatability” and “tooth discoloration” were searched in PubMed until August 2016. A hand search was conducted of the last 2 years’ worth of issues of the following major endodontic and dentistry journals, *International Endodontic Journal*; *Journal of Endodontics*; and *Iranian Endodontic Journal* and *Dental Material Journal*. Additional studies were identified through reference checking that were not found in the electronic search. This process was continued until any new articles were found. Sixty eight articles were finally included in this study and reviewed in detail.

## Results

**Biologic properties:**

***Genotoxicity***

Genotoxicity is an action on cell's genetic material which may influence its integrity. This is one of the most important indicators of carcinogenicity [10, 11]. Genotoxicity of bioceramic-based sealers were found to be less than AH-Plus sealer [12]. Both MTA and calcium silicate based cements were compatible with MG63 cells, and they were not cancer causing agents [10]. Also MTA and Portland cements were found to be not genotoxic and were not able to induce cellular death [13]. Based on the findings of a study, MTA Plus was less genotoxic than MTA Fillapex and RealSeal self-etch sealer [14]. The order of demonstrated toxicity and DNA double-strand breaks using γ-H2AX assay when compared with resin and silicate-based root canal sealers was as following: AH-Plus Jet>MTA Fillapex>iRoot SP>BioRoot RCS [15].

Both AH-Plus and MTA Fillapex caused an increased micronucleus formation (MN assay) -a genotoxicity assay- when compared with control untreated group and white MTA. MTA Fillapex was the most genotoxic material [16].

***Bioactivity***

The tricalcium silicate phase is responsible for the bioactivity displayed by the material [17]. Studies show that MTA Fillapex stimulates mineralization [18] and exhibits bioactivity by stimulating hydroxyapatite nucleation [19]. Results of evaluating MTA Fillapex biocompatibility and bioactivity on osteoblastic cells indicated that this sealer has cytotoxicity effect in the first periods of cells contact [19, 20]. On the other hand, cell viability and ALP enzyme activity had significantly increased for the extended periods [19]. Also mineralized nodules were detected by Alizarin Red staining of human osteoblast-like cell culture (Saos-2), representing the biocompatibility and bioactivity of the material after the setting time [19]. hTGSC (human tooth germ stem cells) differentiation into odontoblast-like cells in DMEM medium was induced with MTA and iRoot SP. In addition, more inductive potential and hard tissue deposition occurred more with MTA than iRoot SP [21]. In addition, AH-Plus and MTA Fillapex sealers induce micronucleus formation (genotoxicity) and cell death [16].

BioRoot RCS has the capacity to induce secretion of significant levels of osteogenic and angiogenic factors such as BMP-2, VEGF, and FGF-2 [22]. 

The Ca and Si incorporation by intertubular dentine may be regarded as an indicator of a material bioactivity. Moreover, the dentine uptake of Ca most probably causes chemical and structural modification of the hard tissue, which leads to the acquirement of higher acid resistance and physical strength [23]. The Si incorporation may also have certain biological significance, because Si is known to enhance the rate of new bone growth when released from bioactive materials *in vivo *[24] and Si induces remineralization of demineralized dentine *in vitro *[25].

ProRoot Endo Sealer in contact with phosphate-containing fluid demonstrates *in vitro* bioactivity [26]. The ProRoot Endo Sealer induce secretion of amorphous calcium phosphate-like phases and apatite-like phases after simulated body fluid storage [27].

Also, calcium phosphate phase was formed in contact with physiologic solution in EndoSequence BC and MTA Fillapex sealers [28]. But, Ca and Si incorporation couldn’t be seen by BC sealer in human root canal dentine [29]. Thus, clinically, material bioactivity cannot be assumed [28].

Although MTA Fillapex is a sealer with the basis of Portland cement, there were any hydration byproducts in the cement matrix and any Portlandite peak on the X-ray diffractogram, which is not in line with EndoSequence BC Sealer [28].

The formation of an interfacial layer at the dentine-material interface had been attributed to the apatite-deposition ability of the silicate-based materials in the presence of phosphate solutions. When hydrated phase and calcium hydroxide (Portlandite) are produced, and the dissociation of this by-product promotes, an increase occurs in the environment pH and provides calcium ions to interact with the phosphates of the surrounding tissues to induce apatite deposition [30]. It has been suggested that dentine may uptake the elements released by bioactive materials, such as calcium and silicon, that leads to an increased mineralization in the adjacent dentine [30].

In human osteoblast cell culture of MTA-Fillapex, after seven days, a significant decrease in cytotoxicity and increase in hydroxy apatite nucleation was seen. When comparing to Epiphany and ZOE sealer, MTA-Fillapex presented the highest percentage of Alizarin red-stained nodules and hydroxyapatite nucleation [19].

ALP activity of iRoot BP Plus was significantly higher than MTA [31]. qRT-PCR indicated that both Bioaggregate and iRoot BP Plus groups were associated with a higher up regulation of mineralization and odontoblastic differentiation-associated gene expressions as compared to MTA group [31].

***Cytokine production***

The inflammatory process is initiated and maintained by up regulation of a network of chemokines (*e.g.*, IL-8) and proinflammatory mediators (*e.g.,* IL-1β and IL-6) that play distinct or shared biological activities [32]. IL-1β and IL-6 are pivotal in periapical disease development, stimulating osteoclastic differentiation and bone resorption as well as contributing to inflammation by inducing synthesis of other cytokines [33].

The sealers of AH-Plus, EndoSequence BC Sealer, EndoSeal and MTA Fillapex showed varying levels of induction for IL-1β, IL-6, and IL-8 [34]. Upregulation of IL-1β and IL-6 expression was indicated in contact with MTA Fillapex compared to the other sealers and the negative control, while IL-1β and IL-6 expression was similar to negative control group in EndoSeal and EndoSequence BC Sealer [34].

**Clinical properties:**

***Possible advantage of single cone technique***

In the single-cone technique, a sealer with adequate physical and chemical properties is required to flow and fill the interfaces between the cone and dentin in order to provide a tight seal [35]. There are several commercial sealers available with different adhesive mechanisms that have been designed to be used in this fashion such as EndoREZ [36], AH-26 [37] and calcium silicate based sealers [35]. These are expected, by interacting with dentinal fluids, to create and deposit intra fibrillar apatite and form tag-like structures within dentin, characterizing their bioactivity [29]. 

A higher linear dislocation resistance and a consequent increase in push-out bond strength are expected when lateral compaction is used compared to single-cone technique for MTA Fillapex and AH-Plus sealers [38].

***Dentinal tubule penetration***

Selected root canal sealer, irrigation procedure and root canal anatomy, could significantly affect the dentinal tubule penetration. 

Use of iRoot SP seems to be advantageous over MTA Fillapex and AH-Plus in dentinal tubule penetration [39], especially when used with continuous wave of condensation rather than single-point technique [40].

***Biomineralization of dentinal tubules***

EndoSeal (Pz-MTA sealer) acts as a bioactive root canal sealer when it was coupled with core material (gutta-percha) and vertical condensation pressure [41]. Intratubular biomineralization depth was significantly enhanced in all Phosphate Buffered Saline (PBS) pretreated canals [41]. However Arias-Moliz and Camilleri [42] suggested not use PBS as final irrigant with BioRoot RCS because of inducing surface changes. They showed that the PBS did not enhance neither the formation of calcium hydroxide nor the antimicrobial activity in contrary to EDTA which opens dentinal tubules and exposes dentine collagen fibrils enhancing the sealers entrapping in dentine structure and reaching the bacteria[42].

Also, the orthograde obturation of root canals with OrthoMTA mixed with PBS may create a favorable environment for bacterial entombment by intratubular mineralization [43]. Zhang *et al*. [44] reported upregulation in mineralization of related genes after application of iRootSP sealer.

***Discoloration of tooth structure***

Because of increasing demands for aesthetics, biomaterials should be chromatically stable, present optical properties similar to dental structures and not exert staining effects to hard dental tissues [45]. When sealer remain confined into pulp chamber, crown discoloration occurs [46]. Recently, it has been shown that both white and gray MTA formulations are able to discolor the tooth [47]. Thus aesthetic test and color objectives are obligatory for every new MTA based material [48]. 

MTA Fillapex, iRoot SP and AH-Plus sealers caused observable tooth discoloration which increased within the first three months and decreased until the 6^th^ month, without any statistically significant difference among them in a study with the sample size of 15 [49]. MTA-Fillapex [48], Endosequence BC [50], AH-Plus [50, 51] and Endoseal [51], led to the least crown discoloration that was clinically undetectable compared to Roth 811 (a ZnO sealer). Perceptible tooth discoloration was made by MTA Fillapex, iRootSP and AH-Plus sealers without any statistically significant difference between them [49]. 

An important issue need to be concerned is discoloration potential of material in contact with sodium hypochlorite irrigant. As stated by Marciano, each material contains bismuth oxide in composition can cause discoloration in contact with sodium hypochlorite [52]. It could be an issue to be concerned about MTA Fillapex and other bismuth oxide containing sealers. In addition internal bleaching was efficient in discoloured teeth induced by MTA Fillapex and AH-26 sealers [53].

***Fracture resistance***

Endodontically treated teeth are supposed to be susceptible to fracture [54]. Therefore, reinforcing the root canal dentin may be a major purpose of root canal therapies [55]. Bortoluzzi *et al.* [56], demonstrated the use of MTA together with metallic post as an intra-radicular reinforcement treatment in order to strengthen teeth in an experimental weakened immature tooth model. Fracture resistance in root-filled single-rooted premolar teeth was increased in Endosequence BC and AH-Plus Jet sealer contrary to Tech BioSealer Endo [57]. iRoot SP together with ActiV GP promising sealer in terms of increasing *in vitro* resistance to the fracture of endodontically treated roots [58]. Contradictory data was published about reinforcement potential of MTA Fillapex. Mandava *et al.* [59], showed that MTA Fillapex didn’t have reinforcement potential. However, Sagsen *et al.* [60] showed the fracture resistance of instrumented root canals was increased when iRoot SP, AH-Plus and MTA Fillapex sealers were used with gutta-percha. They demonstrated lower vertical root fracture resistance of roots treated with MTA Fillapex rather than iRoot SP and AH-26 was found [61]. In contrast, MTA Fillapex showed higher fracture resistance (higher compressive strength values) compared to iRoot SP sealer in another study [62]. 

***Retreatability***

An ideal root canal filling material should easily be removed for retreatment purposes [63]. The conventional retreatment techniques are not able to completely remove Total Fill BC Sealer and MTA Fillapex [64]. Retreatability of EndoSequence BC sealer and AH-Plus sealer were comparable to each other [65]. In addition, retreatability of iRoot SP and AH-Plus are similar [66]. None of the tested iRoot SP, MTA Fillapex, AH-Plus and AH-26 sealers were completely removed from the root canal system during retreatment with rotary systems [63, 67]. Retreatment time to reach the working length was lower for MTA Fillapex than iRoot SP or AH-26 sealers [63]. In addition, MTA Fillapex showed less remaining root filling material than MTA Plus [67]. Chloroform, Endosolv E and Eucalyptol soften gutta-percha and MTA Fillapex sufficiently to aid in re-establishing apical patency during endodontic retreatment [68]. However, MTA Fillapex was not sufficiently dissolved in chloroform and eucalyptoil [69] and chloroform was a more effective solvent than eucalyptol or Endosolv E for MTA Fillapex [70].

When using rotary retreatment systems without solvent, MTA Fillapex showed the least and AH-Plus revealed the most sealer remnants in canals as assessed by cone-beam computed tomography (CBCT) [71].

***Sealer type and post cementation timing***

Limited studies found in respect to timing of fiber post cementation. As, MTA Fillapex was compared to AH-Plus sealer and revealed that immediate or delayed post cementation had no effect on post-dentin adhesiveness [72]. However, the type of sealer was evaluated to be an important issue. As, AH-Plus combined with delayed fiber post insertion resulted in better adhesive properties [72].

***Calcium silicate based sealer fillings with hygro expandable cones (HEC)***

Hygro expandable cones are commercially named C Point or Smart Points and had been introduced in order to enhance integrity of obturating material. However, significant structural defects in them had been confirmed by optical microscopy after physical sectioning [73]. 

***Sealers and warm gutta-percha obturation techniques***

In general, the sealers may reduce the heat generated on the external root surfaces during the heating phase [74]. Chemical structure of some sealers changes after exposure to heat, whilst some other sealers remain unaffected. Contrary to AH-Plus sealer, MTA Fillapex is suitable with warm gutta-percha obturation techniques [75, 76]. Film thickness, flow, setting time and material chemistry of MTA Fillapex were unaffected by heat [75]. MTA Plus is another sealer which has been investigated as not being affected by heat [74].

***Clinical success***

The main goal of endodontic treatment could be clinical success. So, randomized clinical trials with long term follow up and large sample size should be conducted to confirm endodontic sealers clinical success. Only one study [77] was found on the issue with 15 sample size conducted on zinc oxide based, resin-based and MTA mixed with propylene glycol as a sealer and found out similar clinical and pathologic effectiveness of all. However they did not use commercial MTA-based sealers stated above, and despite their small sample size, the results could be helpful.

## Conclusion

On the basis of reviewed literature, it could be assumed that genotoxicity of resin based sealers are higher than calcium silicate based sealers, although both AH-Plus and MTA Fillapex caused an increased micronucleus formation (MN assay) when compared with control untreated group. Also it can be concluded that MTA Fillapex may be associated with acute inflammation after its use during root canal filling which is in contrast to other sealers. Dentinal tubule penetration of iRoot SP was more than MTA Fillapex and AH-Plus. Both resin based and calcium silicate based sealers caused perceptible tooth discoloration. There are controversies about fracture resistance, apical patency and retreatability of different sealers.
